# Concordance between muscle mass assessed by bioelectrical impedance analysis and by dual energy X-ray absorptiometry: a cross-sectional study

**DOI:** 10.1186/s12891-015-0510-9

**Published:** 2015-03-18

**Authors:** Fanny Buckinx, Jean-Yves Reginster, Nadia Dardenne, Jean-Louis Croisiser, Jean-François Kaux, Charlotte Beaudart, Justine Slomian, Olivier Bruyère

**Affiliations:** Department of Public Health, Epidemiology and Health Economics, University of Liège, Avenue de l’Hôpital 3 – CHUB23, 4000 Liège, Belgium; Support Unit in Epidemiology and Biostatistics, University of Liège, Liège, Belgium; Bone Metabolism Department, CHU Liège, Quai Godefroid Kurth 45, 4000 Liège, Belgium; Public Health Department, CHU Liège, Quai Godefroid Kurth 45, 4000 Liège, Belgium; Bone and Cartilage Metabolism Department, CHU Liège, Quai Godefroid Kurth 45, 4000 Liège, Belgium; Department of Motricity Sciences, University of Liège, B21, Allée des Sports, 4000 Liège, Belgium; Department of Physical Medicine and Functional Rehabilitation, CHU of Liège, Bâtiment B 35, 4000 Liège, Belgium

**Keywords:** BIA, Muscle mass, Appendicular lean mass, DXA

## Abstract

**Background:**

Besides magnetic resonance imaging**,** dual energy X-ray absorptiometry (DXA) seems the most reliable tool to evaluate body composition and is often considered as the gold standard in clinical practice. Bioelectrical impedance analysis (BIA) could provide a simpler, portative, and less expensive alternative. Because the body composition assessment by BIA is device-dependent, the aim of this study was to appraise the concordance between the specific bioelectrical impedance device InBody S10 and DXA for the body composition evaluation.

**Methods:**

Body composition, included appendicular lean mass divided by height squared (ALM/ht^2^) was measured by DXA (Hologic QDR Discovery device) and by BIA (InBody S10 Biospace device). Agreement between tools was assessed by means of the Bland Altman method and reliability was determined using the IntraClass Coefficient (ICC). ICC was also computed to assess the reliability of the test-retest performed by the same operator or by two different ones.

**Results:**

A total of 219 subjects were enrolled in this study (mean age: 43.7 ± 19.1 years old, 51.6% of women). For the ALM/ht^2^, reliability of the test-retest of the BIA was high with an ICC of 0.89 (95%CI: 0.86-0.92) when performed by the same operator and an ICC of 0.77 (95%CI: 0.72-0.82) when performed by two different operators. Agreement between ALM/ht^2^ assessed by DXA and BIA was low (ICC = 0.37 (95%CI: 0.25-0.48)). Mean ALM/ht^2^ was 9.19 ± 1.39 kg/m^2^ with BIA and 7.34 ± 1.34 kg/m^2^ with DXA, (p < 0001). A formula developed using a multiple regression analysis, and taking into account muscle mass assessed by BIA, as well as sex and body mass index, explains 89% of the ALM/ht^2^ assessed by DXA.

**Conclusions:**

Although our results show that the measure of ALM/ht^2^ by BIA is reliable, the agreement between DXA and BIA is low. Indeed, BIA seems to overestimate ALM/ht^2^ compared to DXA and, consequently, it is important to use an adapted formula to obtain measurement of the appendicular lean mass by BIA close to that measured by DXA.

## Background

Body composition plays a role in the onset or complications of obesity, type 2 diabetes, and in cardiovascular diseases [1]. From a clinical and epidemiological point of view, the measure of body composition is important to prevent or to treat these diseases.

Different methods have been used to evaluate the total and regional body composition in terms of fat mass, lean soft-tissue mass (comprising muscle, inner organs, and body water), and bone mineral content [2]. One of the most widely used methods is dual energy X-ray absorptiometry (DXA). It is considered valid and reliable, [3] is often referenced in the literature and is regarded as the “gold standard” for this kind of assessment in clinical practice [4-7]. Indeed, it is a safe and non-invasive method that allows not only the evaluation of whole body composition but also of separate body segments, with high precision, reproducibility and accuracy [8]. However, because of the high cost of the equipment, operation and maintenance and its non-portable nature, its use may be limited. To avoid these problems, a bioelectrical approach has been suggested. Indeed, bioelectrical impedance analysis offers an inexpensive and easy to perform alternative to assess body composition [9]. Its reliability has been demonstrated in a few studies but it seems to be device-dependent [10].

One of the major challenges in field research is the difficulty to adequately measure body composition outside the laboratory, where more and more research is now being performed [1]. To this end, a (new) device has recently been introduced on the market: the portable InBody S10 multifrequency body composition analyzer. However, to our knowledge, no study has yet appraised the accuracy of the InBody S10 body composition analyzer in comparison with those obtained with DXA in adult population. It should be noted that DXA is the most frequently used reference device for the validation of BIA devices for body composition. Therefore, the aim of this study was to appraise the concordance between body composition measures, and more specifically appendicular lean mass per height square, obtained from the InBody S10 body composition analyzer and those obtained with dual energy X-ray absorptiometry (DXA).

## Methods

### Study design

This is a cross-sectional analysis performed in an outpatient clinic, in Liège, Belgium. Data collection was conducted from December 2013 until March 2014. The study was approved by the Ethics Committee of the University Hospital of Liège.

### Subjects

Volunteers, adult subjects, were recruited from the community using advertisements in local newspapers and public places. Exclusion criteria, were the contraindications of the device: presence of an electronic implant (heart pacemaker, brain stimulator), body mass index over 50 kg/m^2^, limb amputation, pregnancy. All subjects gave a written informed consent prior to inclusion in the study.

To ensure adequate power, the calculation of the sample size was made before the beginning of the study. The formula used for this calculation is as follows:

$$ \mathrm{n}=\frac{2\sigma 2\left[QG\left(1-\frac{\alpha }{2}\right)+QG\left(1-\beta \right)\right]2}{\varDelta 2} $$

where, σ ^2^ is the variance difference between the result of appendicular lean mass obtained by BIA and the result obtained by DXA in a pilot study (=3.25^2^ kg); α is the type I error = 0.05; β is the type II error = 0.1; Δ ^2^ is the maximum tolerated difference between the results obtained by BIA and by DXA (this was fixed arbitrarily to 1.27 kg, on the basis of the definition of sarcopenia by the European Working Group on Sarcopenia in Older People [11]).

The result of this calculation was n = 138, this meant that a minimum of 138 subjects were required to demonstrate any difference in muscle mass measured by the 2 studied devices. Since the Belgian population (Eurostat 2012) consists of 2.135.600 people aged 18–34 years, 4.504.729 people aged 35–64 years and 1.924.934 people aged 65 years or more, our study had to include respectively 34, 73 and 31 people in each age group in order to respect the age distribution of the population.

### Anthropometric measurements

Height was measured to the nearest 0.1 cm using a stadiometer and weight to the nearest 0.1 kg using a weighting-scale. Abdominal circumference was also measured, to the nearest 0.1 cm, using a tape measure at the navel. Muscle mass, especially appendicular lean mass (ALM), was assessed by the two methods explained below. ALM was calculated as the sum of lean mass of arms and legs [12].

### Bioelectrical Impedance Analysis (BIA)

A multi-frequency bioelectrical impedance analyzer, InBody S10 Biospace device (Biospacte Co, Ltd, Korea/Model JMW140) was used according to the manufacturer’s guidelines. BIA estimates body composition using the difference of conductivity of the various tissues due to the difference of their biological characteristics. Conductivity is proportional to water content, and more specifically to electrolytes, and it decreases as the cell approaches a perfect spherical shape. Adipose tissue is composed of round shaped cell and contains relatively little water compared to other tissues like muscle; therefore conductivity will decrease as body fat increases. In practice, electrodes are placed at 8 precise tactile-points of the body to achieve a multi-segmental frequency analysis. A total of 30 impedance measurements are obtained using 6 different frequencies (1 kHz, 5 kHz, 50 kHz, 250 kHz, 500 kHz, 1000 kHz) at the 5 following segments of the body: right and left arms, trunk, right and left legs.

### Dual energy X-ray Absorptiometry (DXA)

As reference method, DXA scan (Hologic QDR Discovery device, Inc USA) was used for the measurement of whole and regional body composition, including a three-compartment model estimating body composition in terms of fat, bone mineral, and all other fat-free mass that does not include bone. DXA provides thus bone density estimates, and regional estimates of body composition (i.e. parts of the body), by measuring body’s absorbance of X-rays at two different energies using the fact that fat, bone mineral, and fat-free soft tissue have different absorption properties. The subjects were positioned for whole-body scans according to the manufacturer’s protocol. Subject laid in a supine position on the scanner table, with straight-legs and their arms close to the body. They were instructed to remain as still as possible for the duration of the scan. Whole-body composition analysis provided data on different regions of interest, including trunk, arms and legs. The DXA machine was calibrated daily against a phantom spine containing composites of bone, fat and lean tissue supplied by the manufacturer before testing. This procedure has been validated for general DXA use [13].

### Collected data

Other variables were collected to characterize the population.

First, grip strength was assessed by means of a hydraulic dynamometer (Saehan Corporation, MSD Europe Bvba, Belgium, an isometric hydraulic hand dynamometer). According to the American Society of Hand Therapists, this particular instrument can provide the most stable results during repeated gripping trials. Its excellent test-retest reliability has been confirmed in many studies, with obtained IntraClass Correlation Coefficient (ICC) values ranging between 0.81 and 0.98 [14]. In our study, we used the following standardized protocol for the measurement of grip strength [15]. The participant was asked to sit comfortably on a standard chair with legs, back support and fixed arms. He was then advised to squeeze as hard as possible the hand dynamometer for up to six seconds and then relax. Three measurements for each hand, alternating sides, were performed consecutively and without rest. According to Watanabe [16], continuous measurements are not affected by fatigue, especially in the dominant hand. To encourage the subjects to get a score as high as possible, the best of the six grip strength measurements was recorded and later used in statistical analyses, as recently recommended by Roberts [15].

Thereafter, participant’s leisure time activity was evaluated using the short version of the Minnesota Leisure Time Physical Activity Questionnaire. This questionnaire asks participants about types, frequency and duration of their leisure time activity (average hour/day in the following four categories: walking, doing gymnastics or workouts, engaging in sports, and doing household activities). The kcal burned per day was calculated using the activity metabolic index, which allows the calories burned to be measured using the metabolic equivalent of tasks [17,18].

### Statistical analysis

A Shapiro-Wilk test verified the normal distribution for all parameters. Quantitative variables were expressed by mean ± standard deviation (SD), or by median and interquartile range (P25-P75) for asymmetric distributions. Qualitative variables were expressed by number and percentage. Agreement between tools was assessed by means of the Bland Altman method and reliability by means of the IntraClass Correlation Coefficient (ICC) [14]. ICC was also computed to assess the reliability of the test-retest performed by the same operator or by two different ones. The closer the coefficient is to 1, the higher the reliability. We considered an ICC over 0.90 as very high, between 0.70 and 0.89 as high and between 0.50 and 0.69 as moderate [19]. Body composition obtained with the different methods (BIA and DXA) were compared using the t-test or Wilcoxon signed-rank test when appropriate. Analyzes were also performed by gender and by age category (i.e. 18–34 years; 35–64 years and 65+ years).

A multiple regression was conducted to obtain a muscle mass assessed by BIA, close to that measured by DXA. All calculations were performed by using Statistica 10 software, SAS statistical package (version 9.3 for windows) and R statistical packages. Results were considered to be statistically significant at the 5% critical level (p < 0.05).

## Results

A total of 219 subjects (51, 6% of women) were enrolled in this study. The mean age was 43.7 ± 19.1 years. Clinical and demographic characteristics of the subjects are resumed in Table 1.

**Table 1:** **Clinical and demographic characteristics of the subjects**

**Characteristics**		**Mean ± SD**	**Number (Frequency)**
Age (years)		43.7 ± 19.1	
	18-34 years		79 (36.1%)
	35-64 years		92 (42.0%)
	>65 years		48 (21.9%)
Sex			
	Women		113 (51.6%)
	Men		106 (48.4%)
Ethnicity			
	White		216 (98.6%)
	Black		3 (1.4%)
Height (cm)		169.3 ± 11.0	
Weight (kg)		71.9 ± 14.6	
Body mass index (kg/m^2^)		25.7 ± 9.11	
Waist circumference (cm)		77.6 ± 10.2	
Dominant hand			
	Right hand		203 (92.7%)
Grip strength (kg)		40.5 ± 15.0	
Minnesota questionnaire (kcal/day)		493.2 ± 377.2	

For the reliability of the test-retest of the BIA in the total population, the ICC was equal to 0.89 (95%CI: 0.86-0.92) when measures were taken by the same operator and equal to 0.77 (95%CI: 0.72-0.82) when two different operators took the measures. These analyses were performed by age category and by gender and the results are presented in Tables 2 and 3.

**Table 2:** **Intra-observer reliability of the test-retest of the BIA, stratified by age and sex**

		**ICC (95% CI)**
Total population		0.89 (0.86-0.92)
Age category	18-34 years	0.92 (0.88-0.95)
	35-64 years	0.88 (0.82-0.92)
	65 + years	0.82 (0.71-0.90)
Gender	Men	0.86 (0.81-0.91)
	Women	0.67 (0.56-0.76)

**Table 3:** **Inter-observer reliability of the test-retest of the BIA, stratified by age and sex**

		**ICC (95% CI)**
Total population		0.77 (0.72-0.22)
Age category	18-34 years	0.89 (0.83-0.93)
	35-64 years	0.79 (0.70-0.85)
	65 + years	0.60 (0.39-0.75)
Gender	Men	0.52 (0.37-0.65)
	Women	0.65 (0.53-0.75)

**Figure 1:**
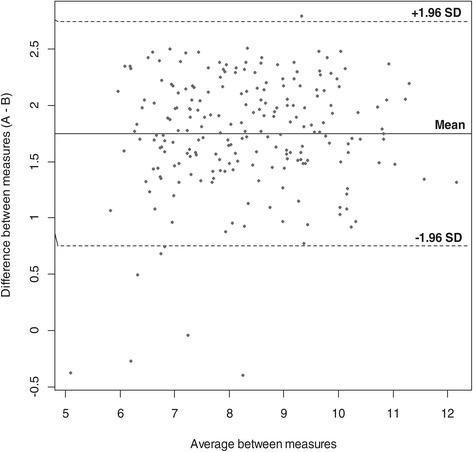
**Bland-Altman plot.**

Analyzes performed in the total population show that agreement between appendicular lean mass assessed by DXA and BIA was low (ICC = 0.37 (95%CI: 0.25-0.48)) (Table 4). Mean appendicular lean mass per height was 9.19 ± 1.39 kg/m^2^ with BIA and 7.44 ± 1.34 kg/m^2^ with DXA (p < .0001). Differences between methods (BIA and DXA) were plotted against the average of the two measurements (Figure 1). The mean difference was equal to 1.75 ± 0.51, with a tendency for BIA to overestimate appendicular lean mass. The inter-individual variation was high, in such an extent that some individuals were out of the limits of agreement ranging from 0.75 to 2.75. These patients were significantly older than patients within the limits (62.0 ± 13.2 vs 43 ± 18.9 years old, p = 0.006). They also had a grip strength significantly lower (24.8 ± 12.4 vs 39.8 ± 14.1 kg, p = 0.004). Moreover, agreement between BIA and DXA was low for lean mass of both the upper limbs and the trunk, as shown in Table 4. However, agreement between BIA and DXA was high for upper limb (95%CI: 0.92 (0.90-0.94) for left arm and 0.87(0.83-0.90) for right arm. Analyses were also stratified for age and sex but of course with a reduced sample size in each subgroup and results are presented in Table 5.

**Table 4:** **Agreement between BIA and DXA for all patients**

**Variables**	**ICC (95% CI)**
Appendicular lean mass/height^2^ (kg/m^2^)	0.37 (0.25 – 0.48)
Lean mass left arm (kg)	0.92 (0.90 – 0.94)
Lean mass right arm (kg)	0.87 (0.83 – 0.90)
Lean mass trunk (kg)	0.82 (0.77 – 0.86)
Lean mass right leg (kg)	0.18 (0.049 – 0.30)
Fat mass (kg)	0.27 (00.15 – 0.39)
Body fat (%)	0.044 (−0.089 – 0.17)

**Table 5:** **Agreement between BIA and DXA, stratified by age and gender**

		**Variables**	**Men ICC (CI 95%)**	**Women ICC (CI 95%)**
Age category	18-34 years	Appendicular lean mass/height square (kg/m^2^)	0.0085 (−0.29-0.30)	−0.43 (−0.66- -0.14)
		Lean mass left arm (kg)	0.92 (085–0.95)	0.45 (0.16-0.67)
		Lean mass right arm (kg)	0.89 (0.80-0.94)	0.58 (0.32-0.75)
		Lean mass trunk (kg)	0.91 (0.84-0.95)	0.44 (0.15-0.67)
		Lean mass left leg (kg)	0.089 (−0.22-0.38)	−0.14 (−0.44-0.18)
		Lean mass right leg (kg)	0.086 (−0.22-0.37)	−0.13 (−0.43-0.19)
		Fat mass (kg)	−0.038 (−0.33-0.26)	−0.086 (−0.39-0.23)
		Body fat (%)	−0.25 (−0.43-0.15)	−0.44 (−0.66-0.15)
	35-64 years	Appendicular lean mass/height square (kg/m^2^)	−0.11 (−0.38-0.18)	−0.038 (−0.031-0.24)
		Lean mass left arm (kg)	0.81 (0.68-0.89)	0.37 (0.11-0.59)
		Lean mass right arm (kg)	0.40 (0.13-0.61)	0.65 (0.46-0.79)
		Lean mass trunk (kg)	0.42 (0.15-0.63)	0.70 (0.53-0.82)
		Lean mass left leg (kg)	−0.0019 (−0.29-0.28)	−0.0046 (−0.29-0.28)
		Lean mass right leg (kg)	0.050 (−0.24-0.33)	−0.045 (−0.32-0.24)
		Fat mass (kg)	0.17 (−0.12-0.44)	0.19(−0.098-0.44)
		Body fat (%)	−0.23 (−0.49-0.057)	−0.19 (−0.45-0.093)
	65+ years	Appendicular lean mass/height square (kg/m^2^)	0.32 (−0.12-0.66)	−0.23 (−0.54-0.13)
		Lean mass left arm (kg)	0.92 (0.80-0.96)	0.64 (0.37-0.81)
		Lean mass right arm (kg)	0.61 (0.25-0.82)	0.85 (0.71-0.92)
		Lean mass trunk (kg)	0.63 (0.28-0.83)	0.60 (0.29-0.77)
		Lean mass left leg (kg)	0.24 (−0.21-0.61)	−0.078(−0.42-0.28)
		Lean mass right leg (kg)	0.33 (−0.12-0.68)	−0.18(−0.50-0.18)
		Fat mass (kg)	0.24 (−0.21-0.60)	0.72 (0.49-0.85)
		Body fat (%)	−0.14 (−0.53-0.31)	0.42 (0.08-0.67)

Using a multiple regression analysis, the following equation has been developed to obtain an appendicular lean mass value by BIA closed to that measured by DXA: ALM/ht^2^ (DXA) = 0.04*BMI – 0.58*Women + 0.69*ALM/ht^2^ (BIA) (p < 0.0001; R^2^ = 0.89) (Table 6).

**Table 6:** **Multiple regression**

**N = 217**			
**R** ^**2**^ **= 0.89**			
**P < .0001**			
**Variables**	**β**	**Error type β**	**p-value**
Intercept	0.41	0.39	.28
Body mass index	0.04	0.01	<.0001
Sex	−0.58	0.095	.0001
Appendicular lean mass/height ^2^	0.69	0.04	.0001
Age	0.002	0.002	.46
Abdominal circumference	−0.009	0.004	.81

## Discussion

It is important for clinical practice and epidemiological research to develop simple and accurate devices for the measurement of body composition. Therefore we investigated the concordance between body composition evaluations achieved with the new portable InBody S10 body composition analyzer and the “gold standard” DXA. Our results show a good reliability of BIA to assess ALM/ht^2^, in agreement with the literature [20,21] which considers BIA as a reliable method. This seems true, whatever the age category. The BIA method also seems to have a high degree of appropriateness (relevant aspects are measured, there is a clear endpoint at the evaluation), [21] constituting a promising assessment tool.

However, in line with results of others studies, [19,22,23] we showed that agreement between ALM/ht^2^ square obtained by BIA and DXA is low, with an ICC of 0.37. Indeed, we found that, compared to DXA, BIA seems to overestimate ALM/ht^2^ by 1.75 kg on average. Several studies also showed that, compared to DXA, BIA has a tendency to overestimate muscle mass [23-25]. A study, recently published [25], showed a difference of 1.5 kg between appendicular muscle mass divided by height square observed by BIA and by DXA, which is slightly lower than our results (i.e. 1.75 kg), and could be explained by the particular bioelectrical impedance device used. According to our results, it seems that, when assessing lean mass, agreement between BIA and DXA is high for the upper limbs and low for the lower limbs. Also, as suggested by several studies [26], agreement between BIA and DXA is low for fat mass. These studies have used various bioelectrical impedance devices and admit that the BIA tends to underestimate fat mass and overestimate lean mass. Indeed, differences between BIA and DXA are most likely dependent on the device used. To the best of our knowledge, this is the first study that compares values of appendicular muscle mass obtained by the BIA InBody S10 to those obtained by DXA. Indeed, many bioelectrical impedance devices exist (Tanita, InBody720, BIA 101 Q-RJL system,…) and accuracy of each ones probably differs. This might explain why we observed a difference of 1.75 kg between ALM/ht^2^ by BIA and by DXA while, as described above, other researchers obtained different values. Another explanation could be that measurements have been carried out in different populations.

Karelis [1] validated another type of InBody, the InBody S230 device, compared to DXA. Although a systematic bias for the estimation, in men and women, of trunk and appendicular lean mass is present in this study, using the InBody S230, it seems that their results regarding muscle mass are quite similar to ours. However, unlike our study with the InBody S10, their study seems to show a good correlation for fat mass and body fat between their BIA device and DXA. In view of these results, it seems that the estimation of body composition depends on the device used although some similarities between marks can be observed.

Therefore, we suggest a formula taking into account muscle mass assessed by BIA (InBody S10), as well as sex and body mass index, which explains 89% of the muscle mass assessed by DXA. A similar equation was developed in older community-dwelling Korean adults [27]. This last study presents an r^2^ identical to that reported in this present paper (r^2^ = 0.89). Indeed, it seems quite clear that population-specific equations and devices specific equations are necessary to obtain the most accurate estimates of body composition by BIA [10].

The present study has both strengths and weaknesses. The main strength is that the study included a large number of representative individuals in terms of age, of the Belgian population. Then, because age and gender related differences in body compositions could modify the results obtained, we performed per sex and per age analyses to eliminate these confounding factors. The study has also several limitations. First, we only studied Belgian subjects. Studies of other ethnic groups, children or adolescents could yield different results. Different results could also be obtained between athletes and sedentary subjects. Second, the hydration status of the study subjects was not determined before the body composition assessment and could influence the results. Third, the results are only applicable for the InBody S10 device. Future research should be conducted to measure the validity and reliability of other type of BIA. At last, analyzes carried out by age and gender groups should be interpreted with caution because the sample size is greatly reduced in some of these subgroups.

## Conclusion

In conclusion, our results show that the measure of muscle mass by BIA, using the InBody S10, is reproducible, when performed by the same operator and when performed by two different ones. Nevertheless, the concordance between muscle mass measured by BIA and by DXA is low. Indeed, BIA seems to overestimate muscle mass compared to DXA. Consequently, it is important to use a formula to obtain an adapted muscle mass value by BIA close to that measured by DXA to make of the BIA a portable and easy to use alternative to DXA.

## Abbreviations

BIA: Bioelectrical impedance analysis
DXA: Dual energy x-ray absorptiometry
ALM/ht^2^: Appendicular lean mass divided by height squared
ICC: IntraClass coefficient
BMI: Body mass index
